# Complete genome sequence of the filamentous gliding predatory bacterium *Herpetosiphon aurantiacus* type strain (114-95^T^)

**DOI:** 10.4056/sigs.2194987

**Published:** 2011-12-23

**Authors:** Hajnalka Kiss, Markus Nett, Nicole Domin, Karin Martin, Julia A. Maresca, Alex Copeland, Alla Lapidus, Susan Lucas, Kerrie W. Berry, Tijana Glavina Del Rio, Eileen Dalin, Hope Tice, Sam Pitluck, Paul Richardson, David Bruce, Lynne Goodwin, Cliff Han, John C. Detter, Jeremy Schmutz, Thomas Brettin, Miriam Land, Loren Hauser, Nikos C. Kyrpides, Natalia Ivanova, Markus Göker, Tanja Woyke, Hans-Peter Klenk, Donald A. Bryant

**Affiliations:** 1 DOE Joint Genome Institute, Walnut Creek, California, USA; 2 Los Alamos National Laboratory, Bioscience Division, Los Alamos, New Mexico, USA; 3 Leibniz Institute for Natural Product Research and Infection Biology, Hans Knöll Institute, Jena, Germany; 4 The Pennsylvania State University, University Park, Pennsylvania, 16802 USA; 5 Current address: Dept. of Civil and Environmental Engineering, University of Delaware, Newark, Delaware, 19716 USA; 6 Oak Ridge National Laboratory, Oak Ridge, Tennessee, 37821 USA; 7 Leibnitz Institute DSMZ – German Collection of Microorganisms and Cell Cultures, Braunschweig, Germany

**Keywords:** Chemoorganoheterotrophic, Gram-negative, gliding, ensheathed filaments, free-living, predator, *Herpetosiphonaceae*, *Chloroflexi*, DOEM2005

## Abstract

*Herpetosiphon aurantiacus* Holt and Lewin 1968 is the type species of the genus *Herpetosiphon*, which in turn is the type genus of the family *Herpetosiphonaceae*, type family of the order *Herpetosiphonales* in the phylum *Chloroflexi*. *H. aurantiacus* cells are organized in filaments which can rapidly glide. The species is of interest not only because of its rather isolated position in the tree of life, but also because *Herpetosiphon* ssp. were identified as predators capable of facultative predation by a *wolf pack* strategy and of degrading the prey organisms by excreted hydrolytic enzymes. The genome of *H. aurantiacus* strain 114-95^T^ is the first completely sequenced genome of a member of the family *Herpetosiphonaceae*. The 6,346,587 bp long chromosome and the two 339,639 bp and 99,204 bp long plasmids with a total of 5,577 protein-coding and 77 RNA genes was sequenced as part of the DOE Joint Genome Institute Program DOEM 2005.

## Introduction

Strain 114-95^T^ (= ATCC 23779 = DSM 785 = CCUG 48726) is the type strain of *Herpetosiphon aurantiacus*, which in turn is the type species of the genus *Herpetosiphon* [1,2]. Because most of the species were reclassified as members of other genera in 1998 [3], only one other species currently remains in this genus: *H. geysericola* (Copeland 1936) Lewin 1970. The genus name, meaning gliding tube, was derived from the Greek words *herpeton*, gliding animal or reptile, and *siphon*, tube or pipe [4]. The species epithet is derived from the Neo-Latin adjective *aurantiacus*, meaning orange-colored [4]. Strain 114-95^T^ was originally isolated from the slimy coating of a freshwater alga (*Chara* sp.) in Birch Lake, Minnesota (USA), but strains belonging to the species were also isolated from well water, cow dung, hot springs and marine shores [1]. *H. aurantiacus* 114-95^T^ is capable of predation of other bacteria and can thereby destroy whole colonies [5]. It has even been suggested that *Herpetosiphon* spp. are capable of facultative predation by a *wolf pack* strategy, in which a quorum of predatory cells is required to degrade the prey organism by excreted hydrolytic enzymes [6]. Here we present a summary classification and a set of features for *H. aurantiacus* 114-95^T^, together with the description of the complete genome and its annotation.

## Classification and features

A representative genomic 16S rRNA sequence of *H. aurantiacus* strain 114-95T was compared using NCBI BLAST [7,8] under default settings (e.g., considering only the high-scoring segment pairs (HSPs) from the best 250 hits) with the most recent release of the Greengenes database [9] and the relative frequencies of taxa and keywords (reduced to their stem [10]) were determined, weighted by BLAST scores. The most frequently occurring genera were *Herpetosiphon* (82.9%), *Chloroflexus* (9.9%), '*Kouleothrix*' (4.5%), *Oscillochloris* (2.2%) and '*Chlorothrix*' (0.5%) (46 hits in total). Regarding the 13 hits to sequences from members of the species, the average identity within HSPs was 99.8%, whereas the average coverage by HSPs was 96.9%. Regarding the two hits to sequences from other members of the genus, the average identity within HSPs was 97.8%, whereas the average coverage by HSPs was 94.2%. Among all other species, the one yielding the highest score was *Herpetosiphon geysericola* (NR_028694), which corresponded to an identity of 97.8% and an HSP coverage of 94.2%. (Note that the Greengenes database uses the INSDC (= EMBL/NCBI/DDBJ) annotation, which is not an authoritative source for nomenclature or classification.) The highest-scoring environmental sequence was JF098937 ('skin popliteal fossa clone ncd1008d03c1'), which showed an identity of 98.2% and an HSP coverage of 88.3%. The most frequently occurring keywords within the labels of environmental samples that yielded hits were 'soil' (4.2%), 'microbi' (4.1%), 'geyser' (3.3%), 'geotherm' (2.9%) and 'mat' (2.8%) (204 hits in total). Environmental samples that yielded hits of a higher score than the highest scoring species were not found. These keywords fit well with the ecological properties reported for strain 114-95^T^ in the original description [1].

**Figure 1:**
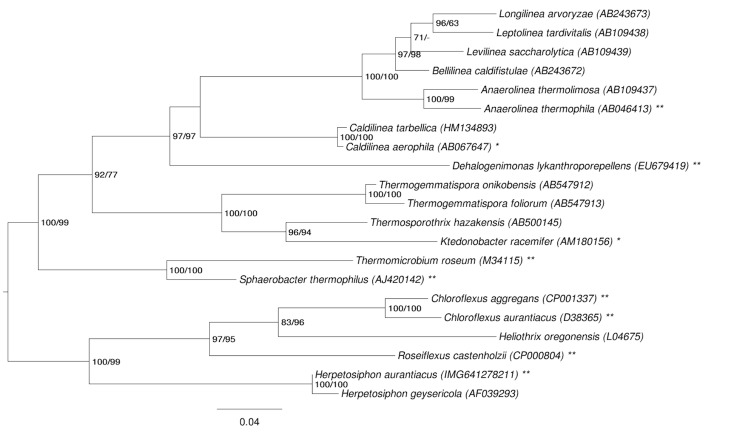
Phylogenetic tree highlighting the position of *H. aurantiacus* relative to the other type strains within the phylum *Chloroflexi*. The tree was inferred from 1,350 aligned characters [11,12] of the 16S rRNA gene sequence under the maximum likelihood (ML) criterion [13]. Rooting was done initially using the midpoint method [14] and then checked for its agreement with the current classification (Table 1). The branches are scaled in terms of the expected number of substitutions per site. Numbers adjacent to the branches are support values from 100 ML bootstrap replicates [15] (left) and from 1,000 maximum parsimony bootstrap replicates [16] (right) if the value is larger than 60%. Lineages with type strain genome sequencing projects registered in GOLD [17] are labeled with one asterisk, and those also listed as 'Complete and Published' with two asterisks (see [18,19] and AP012029 for *Anaerolinea thermophila*, CP002084 for *Dehalogenimonas lykanthroporepellens*, CP001337 for *Chloroflexus aggregans*, CP000909 *C. aurantiacus*, and CP000804 for *Roseiflexus castenholzii*).

Figure 1 shows the phylogenetic neighborhood of *H. aurantiacus* in a tree based upon 16S rRNA. The sequences of the five 16S rRNA gene copies in the genome differ from each other by up to two nucleotides, and differ by up to seven nucleotides from the previously published 16S rRNA sequence (M34117), which contains 64 ambiguous base calls.

**Table 1:** Classification and general features of *H. aurantiacus* 114-95^T^ according to the MIGS recommendations [20] and the NamesforLife database [21].

**MIGS ID**	**Property**	**Term**	**Evidence code**
	Current classification	Domain *Bacteria*	TAS [22]
		Phylum *Chloroflexi*	TAS [23,24]
		Class *Chloroflexi*	TAS [23,24]
		Order *Herpetosiphonales*	TAS [25]
		Family *Herpetosiphonaceae*	TAS [26]
		Genus *Herpetosiphon*	TAS [1,2,27]
		Species *Herpetosiphon aurantiacus*	TAS [1,2]
		Type strain 114-95	TAS [1]
	Gram stain	negative	TAS [1]
	Cell shape	cylindrical in unbranched sheathed filaments	TAS [1]
	Motility	gliding	TAS [1]
	Sporulation	not reported	
	Temperature range	not reported	
	Optimum temperature	about 30 °C	NAS
	Salinity	not reported	
MIGS-22	Oxygen requirement	oxic	NAS
	Carbon source	probably carbohydrates	TAS [1]
	Energy metabolism	chemoorganoheterotroph	TAS [1]
MIGS-6	Habitat	diverse: coating of *Chara* sp., lake and well water, cow dung, hot springs, marine shores	TAS [1]
MIGS-15	Biotic relationship	free living	NAS
MIGS-14	Pathogenicity	none known	NAS
	Biosafety level	1	TAS [28]
	Isolation	slimy coating of *Chara* sp.	TAS [1]
MIGS-4	Geographic location	Birch Lake, Minnesota, USA	TAS [1]
MIGS-5	Sample collection time	1961	NAS
MIGS-4.1	Latitude	45.04	
MIGS-4.2	Longitude	94.42	
MIGS-4.3	Depth	not reported	
MIGS-4.4	Altitude	390 m	NAS

Evidence codes - TAS: Traceable Author Statement (i.e., a direct report exists in the literature); NAS: Non-traceable Author Statement (i.e., not directly observed for the living, isolated sample, but based on a generally accepted property for the species, or anecdotal evidence). These evidence codes are from the Gene Ontology project [29].

**Figure 2:**
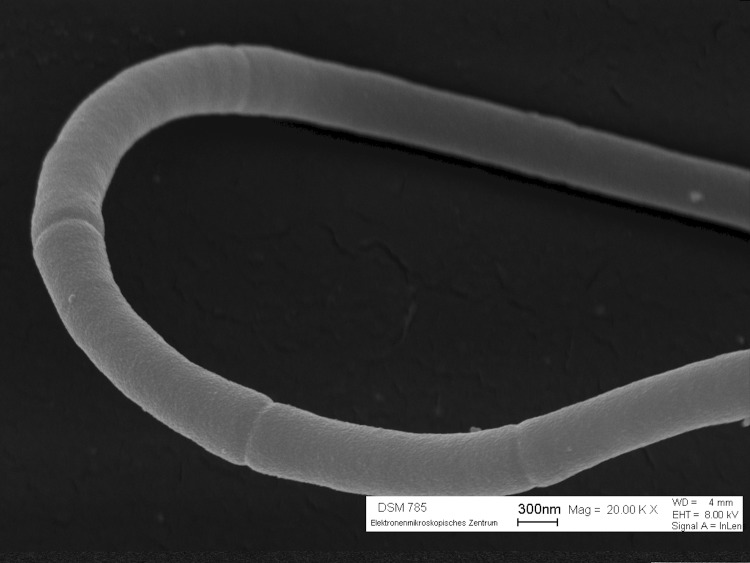
Scanning electron micrograph of a multicellular filament of *H. aurantiacus* 114-95^T^.

Cells of *H. aurantiacus* strain 114-95^T^ are cylindrical measuring 1-1.5 μm by 5-10 μm (Figure 2) [1]. Cells are organized in sheathed filaments of 500 μm length or more [1]. However, the existence of a sheath in the classical sense has been questioned in an analysis of the fine structure of the cells [30]. Cells of strain 114-95^T^ stain Gram-negative, are not flagellated but are motile *via* gliding and divide by the formation of a transverse septum [1]. Colonies are flat, spreading, and rough, and produce an orange pigment [1]. Pigment analyses of a related strain, *H. giganteus* Hp a2, showed that this strain produces γ-carotene, as well as glycosylated and acyl-glycosylated derivatives of 1′-hydroxy-4-keto-gamma-carotene [31]. Strain 115-95^T^ is catalase-positive and hydrolyzes starch, gelatine, casein and tributyrin but not cellulose [1].

### Chemotaxonomy

Data on the structure of the cell wall, quinones, cellular and polar lipids of strain 114-95^T^ are not available, although *H. giganteus* Hp a2 was reported to produce menaquinones 6 and 7 [31]. Members of the *Chloroflexi* do not contain a lipopolysaccharide-containing outer membrane and the peptidoglycan is a variant that usually contains L-ornithine as the diamino acid [32].

## Genome sequencing and annotation

### Genome project history

This organism was selected for sequencing as part of the DOE Joint Genome Institute Program DOEM 2005. The genome project is deposited in the Genomes On Line Database [17] and the complete genome sequence is deposited in GenBank. Sequencing, finishing and annotation were performed by the DOE Joint Genome Institute (JGI). A summary of the project information is shown in Table 2.

**Table 2:** Genome sequencing project information

**MIGS ID**	**Property**	**Term**
MIGS-31	Finishing quality	Finished
MIGS-28	Libraries used	Three genomic Sanger libraries: 3 kb pUC, 8 kb pMCL200 and fosmid pcc1Fos libraries.
MIGS-29	Sequencing platforms	ABI3730
MIGS-31.2	Sequencing coverage	11.0 × Sanger
MIGS-30	Assemblers	phrap
MIGS-32	Gene calling method	Prodigal, GenePRIMPCritica
	INSDC ID	CP000875 (chromosome) CP000876 (plasmid HAU01) CP000877 (plasmid HAU02)
	GenBank Date of Release	November 13, 2007
	GOLD ID	Gc00677
	NCBI project ID	16523
	Database: IMG	2508501111
MIGS-13	Source material identifier	DSM 785
	Project relevance	Biotechnology

### Strain history

The history of strain 114-95^T^ originates with J. G. Holt, who deposited the strain in the ATCC collection in 1961, from which it was distributed to the DSMZ and the CCUG [33].

### Growth conditions and DNA isolation

*H. aurantiacus* strain 114-95^T^ (DSM 785) was obtained from the German Collection of Microorganisms and Cell Cultures (DSMZ). Cells for DNA isolation were grown at 28 °C in the recommended CY liquid medium under oxic conditions with gentle shaking. DNA was isolated by the cetyl trimethylammonium bromide protocol recommended and described by the Joint Genome Institute [34]. The purity, quality and size of the bulk gDNA preparation were assessed according to DOE-JGI guidelines [34] and were consistent with JGI quality-control standards.

### Genome sequencing and assembly

The genome was sequenced using a combination of 3 kb, 8 kb and fosmid DNA libraries. All general aspects of library construction and sequencing can be found at the JGI website [34]. The draft assembly contained 160 contigs in 51 scaffolds. The Phred/Phrap-/Consed software package was used for sequence assembly and quality assessment [35]. Possible mis-assemblies were corrected with Dupfinisher [36]. Gaps between contigs were closed by editing in Consed, custom priming, or PCR amplification. A total of 3,856 additional reactions and two shatter libraries were needed to close gaps and to raise the quality of the finished sequence. The error rate of the completed genome sequence is less than 1 in 100,000. Together, all libraries provided 11.0 × coverage of the genome. There are 85,815 total reactions in the final assembly.

### Genome annotation

Genes were identified using Prodigal [37] as part of the Oak Ridge National Laboratory genome annotation pipeline, followed by a round of manual curation using the JGI GenePRIMP pipeline [38]. The predicted CDSs were translated and used to search the National Center for Biotechnology Information (NCBI) nonredundant database, UniProt, TIGRFam, Pfam, PRIAM, KEGG, COG, and InterPro databases. Additional gene prediction analysis and functional annotation was performed within the Integrated Microbial Genomes Expert Review (IMG-ER) platform [39]

## Genome properties

**Figure 3:**
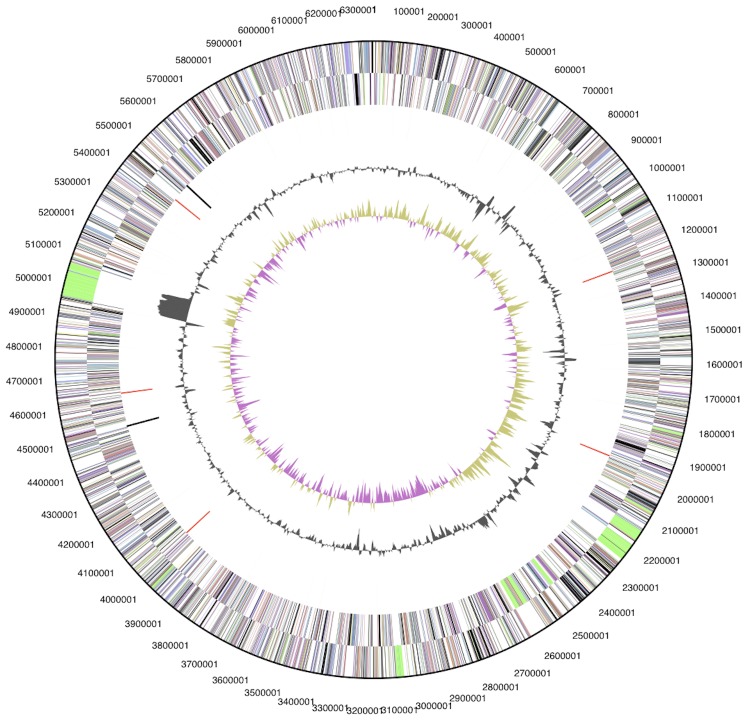
Graphical circular map of the chromosome (not drawn to scale with plasmids). From outside to the center: Genes on forward strand (color by COG categories), Genes on reverse strand (color by COG categories), RNA genes (tRNAs green, rRNAs red, other RNAs black), GC content, GC skew.

**Figure 4:**
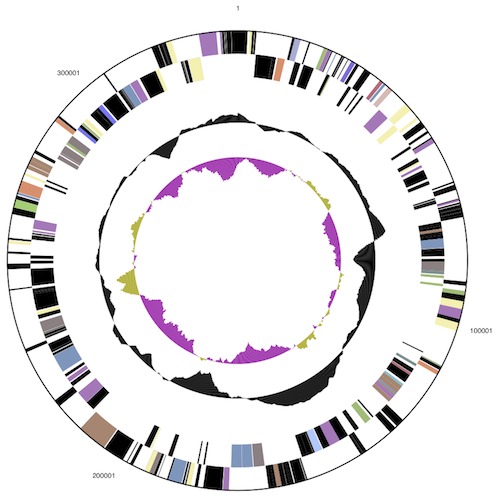
Graphical circular map of the plasmid pHAU01. From outside to the center: Genes on forward strand (color by COG categories), Genes on reverse strand (color by COG categories), RNA genes (tRNAs green, rRNAs red, other RNAs black), GC content, GC skew.

**Figure 5:**
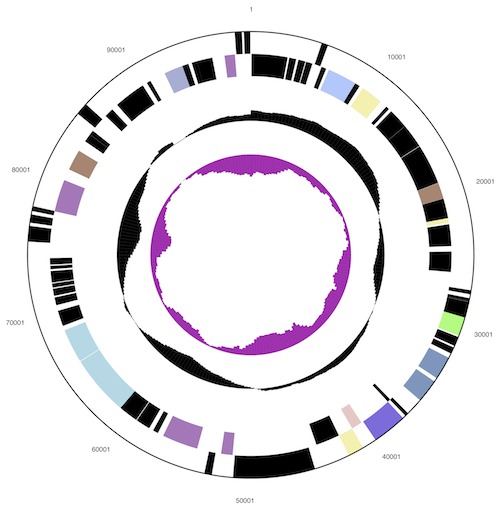
Graphical circular map of the plasmid pHAU02 (not drawn to scale with chromosome). From outside to the center: Genes on forward strand (color by COG categories), Genes on reverse strand (color by COG categories), RNA genes (tRNAs green, rRNAs red, other RNAs black), GC content, GC skew.

**Figure 6:**
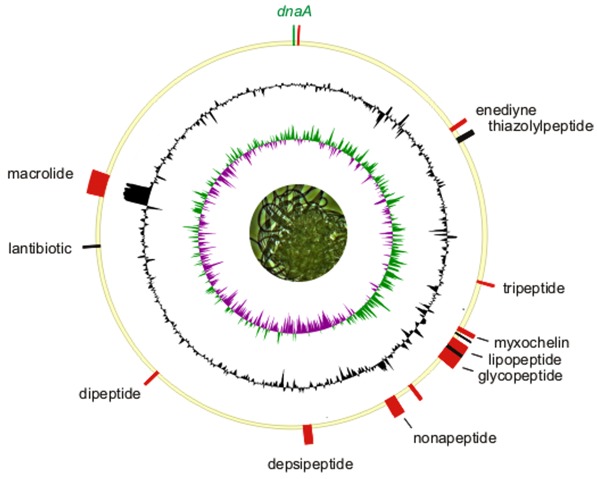
Chromosome of *H. aurantiacus* strain 114-95^T^ oriented to the *dnaA* gene (top). The inner ring shows a normalized plot of GC skew, while the center ring shows a normalized plot of GC content. The outer circle shows the distribution of secondary metabolite gene clusters. Biosynthetic gene clusters associated with thiotemplate-based assembly (PKS, NRPS) are depicted in red and bacteriocin loci are marked in black.

The genome consists of a circular chromosome (6,346,587 bp) and two circular plasmids, pHAU01 (339,639 bp) and pHAU02 (88,204 bp), respectively with an overall G+C content of 50.9% (Table 3, Figure 3, Figure 4, Figure 5 and Figure 6). These are the only plasmids that have been identified to date among the seventeen *Chloroflexi* strains whose genomes have been sequenced. Interestingly, the GC content of the chromosome (50.7%) is notably lower than that of pHAU01 (53.7%) or pHAU02 (53.1%). Plasmid pHAU02 is predicted to encode 71 proteins, and pHAU01 is predicted to encode 231 potential proteins, which included a variety of transposases, phage recombinases and integrases, a CRISPR and CRISPR-associated gene cluster, and a variety of predicted transcription regulators. Neither plasmid encodes a product known to be essential for cell viability. Of the 5,654 genes predicted, 5,577 were protein-coding genes, and 77 encoded RNAs; 82 pseudogenes were identified. The majority of the protein-coding genes (67.4%) were assigned a putative function while the remaining ones were annotated as hypothetical proteins. The distribution of genes into COGs functional categories is presented in Table 4.

**Table 3:** Genome Statistics

**Attribute**	**Value**	**% of Total**
Genome size (bp)	6,785,430	100.00%
DNA coding region (bp)	5,654,495	88.21%
DNA G+C content (bp)	3,453,669	50.90%
Number of replicons	3	
Extrachromosomal elements	2	
Total genes	5,654	100.00%
RNA genes	77	1.36%
rRNA operons	5	
Protein-coding genes	5,577	98.64%
Pseudogenes	82	
Genes with function prediction	3,812	67.42%
Genes in paralog clusters	505	8.93%
Genes assigned to COGs	3,831	67.76%
Genes assigned Pfam domains	3,766	66.61%
Genes with signal peptides	1,071	18.94%
Genes with transmembrane helices	1,495	26.44%
CRISPR repeats	11	

**Table 4:** Number of genes associated with the general COG functional categories

Code	value	%age	Description
J	176	4.1	Translation, ribosomal structure and biogenesis
A	3	0.1	RNA processing and modification
K	370	8.7	Transcription
L	213	5.4	Replication, recombination and repair
B	2	0.1	Chromatin structure and dynamics
D	35	0.8	Cell cycle control, cell division, chromosome partitioning
Y	0	0.0	Nuclear structure
V	93	2.2	Defense mechanisms
T	393	9.2	Signal transduction mechanisms
M	261	6.1	Cell wall/membrane/envelope biogenesis
N	14	0.3	Cell motility
Z	0	0.0	Cytoskeleton
W	0	0.0	Extracellular structures
U	56	1.3	Intracellular trafficking and secretion, and vesicular transport
O	142	3.3	Posttranslational modification, protein turnover, chaperones
C	205	4.8	Energy production and conversion
G	276	6.5	Carbohydrate transport and metabolism
E	307	7.2	Amino acid transport and metabolism
F	92	2.2	Nucleotide transport and metabolism
H	174	4.1	Coenzyme transport and metabolism
I	128	3.0	Lipid transport and metabolism
P	172	4.0	Inorganic ion transport and metabolism
Q	143	3.4	Secondary metabolites biosynthesis, transport and catabolism*
R	647	15.1	General function prediction only
S	349	8.2	Function unknown
-	1,823	32.2	Not in COGs

* manual annotation yielded 6.4% genes in the secondary metabolites biosynthesis, transport and catabolism category.

## Insights into the genome

The phylum *Chloroflexi* is fascinatingly diverse and includes chlorophototrophs (*Chloroflexales*) as well as Gram-positive spore-forming organisms (*Ktedonobacteria*), reductive dehalogenating bacteria (*Dehalococcoidetes*), obligately aerobic heterotrophic thermophiles (*Thermomicrobia*), obligately anaerobic heterotrophs (*Anaerolineae, Caldilineae*), and marine organisms for which no example has yet been cultivated (e.g., SAR202 cluster). *H. aurantiacus* is described as an aerobic heterotroph from the order *Herpetosiphonales*, one of two orders within the class *Chloroflexi*. All characterized members of the other order, *Chloroflexales*, are chlorophototrophs. These include organisms from the genera *Chloroflexus, Roseiflexus, Oscillochloris, Chloronema, Chlorothrix*, and *Heliothrix* [40]. An organism that appears to be most similar although not very closely related to members of *Anaerolinea* has also recently been shown to be chlorophototrophic [41]. The genome sequence strongly supports the conclusion that *H. aurantiacus* is not a chlorophyll-based phototroph or an autotroph. Other than genes for carotenoid biosynthesis, a trait that is very widely distributed among members of all three kingdoms of life, no genes specifically used for photosynthetic light harvesting, electron transport, or chlorophyll biosynthesis were identified in the *H. aurantiacus* genome. Moreover, although *Chloroflexus* spp. and *Roseiflexus* spp. possess the genetic capacity to fix bicarbonate/CO_2_ by the 3-hydroxypropionate cycle, the corresponding genes are absent in *H. aurantiacus* [40,42].

Based upon the genes for carotenogenesis that are found in the *H. aurantiacus* genome, one can predict potential carotenoids that might be produced by this organism. The genome encodes homologs of *crtB* (phytoene synthase, *crtI* (phytoene desaturase), *crtO* (carotene 4-ketolase), *crtD* (hydroxyneurosporene dehydrogenase) and *cruA* (lycopene cyclase), and this combination of enzymes should allow this organism to produce γ-carotene derivatives with a keto-group at the 4 position. Additionally, the genome encodes *cruF* (γ-carotene 1′,2′-hydratase), *cruC* (1′-OH glycosyltransferase), and *cruD*, (acyl transferase) [43-45]. The presence of these genes would allow *H. aurantiacus* to synthesize monocyclic carotenoids carrying glycosyl, or glycosyl-fatty acyl ester moieties, attached to the 1′-OH group at the ψ–end of the molecule. This biosynthetic potential is in good agreement with the carotenoid contents of *H. giganteus* strain Hpa2 [31] and *Roseiflexus* spp. strains. The former produces γ-carotene and its glycosyl and fatty acyl-glycoside ester derivatives, while the latter produce γ-carotene, 4-keto-myxocoxanthin-glucoside, and 4-keto-myxocoxanthin-glucoside fatty acyl esters [44,46].

Scanning of the *H. aurantiacus* genome for siderophore biosynthetic genes revealed the presence of a cluster with remarkable similarity to that of the myxobacterial iron chelator myxochelin [47]. The annotated locus (Haur_01919 – Haur_01928) contains a complete set of genes for the production of 2,3-dihydroxybenzoic acid, including open reading frames encoding isochorismate synthase (Haur_01921), isochorismate hydrolase (Haur_01923) and 2,3-dihydro-2,3-dihydroxybenzoate dehydrogenase (Haur_01919). Additional genes in the operon are proposed to be involved in siderophore transport and iron utilization. Direct comparison with the myxochelin (*mxc*) cluster from *Myxococcus xanthus* DK1622 reveals the absence of an aminotransferase gene analogous to *mxcL*. Since *mxcL* is crucial for the formation of myxochelin B [47], *H. aurantiacus* probably only produces the biosynthetic predecessor mxyochelin A for iron sequestration. Homologs of genes involved in gliding motility in *Myxococcus xanthus* [48,49] and *Flavobacterium johnsoniae* [50-53] were not found in the *H. aurantiacus* genome. The genes required for gliding in *H. aurantiacus* and other members of the *Chloroflexi* have not yet been identified.

One of the most interesting and unique findings within the *H. aurantiacus* genome was the abundance of gene loci involved in secondary metabolism. *Chloroflexi* genomes were screened via BLASTP alignment against a representative library of conserved biosynthetic enzymes, a method proven useful in the analysis of the *Salinispora tropica* genome [54]. Apart from genes involved in fatty acid and carotenoid biosynthesis, no hits were obtained for the chlorophototrophic *Chloroflexi* (Table 5). On the other hand, families of genes encoding enzymes of secondary metabolism were found to be highly overrepresented in the *H. aurantiacus* genome. A total of fourteen biosynthetic gene clusters were identified and annotated, 11 of which are specific for this organism (Table 6). The combined length of these clusters is estimated to be ~448.6 kb, *i.e.* 6.6% of the *H. aurantiacus* genome is dedicated to natural product assembly. The biosynthetic capacity for secondary metabolite production of *H. aurantiacus* is thus comparable to that of other specialist producers of secondary metabolites, such as *Actinobacteria* and *Myxobacteria* [55,56]. The distribution of the biosynthetic loci on the *H. aurantiacus* chromosome seems non-random, with seven of them being located between 1.8 and 2.7 Mb clockwise from the replication origin, i.e. 50% of all clusters are concentrated in a region covering only 14% of the chromosome.

**Table 5:** *Chloroflexi* genome data and biosynthetic potential.

**Organism**	**Size, Mb**	**Topology**	**Biosynthetic Gene Cluster**			
			**PKS**	**NRPS**	**PKS/NRPS**	**Bacteriocin**
*H. aurantiacus* strain 114-95^T^	6.35^1^	Circular	2	4	5	3
*Chloroflexus aurantiacus* J-10-fl *Chloroflexus aggregans* DSM 9485 *Roseiflexus castenholzii* DSM 13941 *Roseiflexus* sp. RS-1	5.26 4.68 5.72 5.80	Circular Circular Circular Circular	- - - -	- - - -	- - - -	- - - -
						

^1^chromosome only

**Table 6:** *Herpetosiphon aurantiacus* strain 114-95^T^ biosynthetic loci.

**Type #**	**Location on the chromosome**	**Features**	**Product**	**Estimated Size [kb]**
**PKS/NRPS** 1	Haur_01976- Haur_01989	2 PKS modules, 6 NRPS modules	Lipopeptide	47.7
2	Haur_02003 Haur_02024	1 PKS module, 11 NRPS modules, genes for the production of hydroxyphenylglycine	Glycopeptide	69.0
3	Haur_02147	1 PKS module, 1 NRPS module	Unknown	12.3
4	Haur_02568-Haur_02581	2 PKS modules, 3 NRPS modules	Depsipeptide	35.9
5	Haur_04184-Haur_04206	16 PKS modules, 7 NRPS modules, genes for the production of hydroxymalonyl-acyl carrier protein	Macrolide	112.6
				
**PKS** 1	Haur_00021	naringenin-chalcone synthase (type-III PKS)	Aromatic polyketide	1.1
2	Haur_00919-Haur_00937	iterative type-I PKS	Enediyne	27.5
				
**NRPS** 1	Haur_01684-Haur_01687	3 NRPS modules	Tripeptide	10.0
2	Haur_01919-Haur_01928	1 NRPS module	Myxochelin	14.0
3	Haur_02229-Haur_02262	9 NRPS modules	Nonapeptide	62.6
4	Haur_03313-Haur_3318	2 NRPS modules	Dipeptide	13.3
**Bacteriocin** 1 2 3	Haur_00966-Haur_00988 Haur_01990-Haur_02002 Haur_03953-Haur_03957	genes for Ser/Thr dehydration and heterocycle formation LanM-like lanthionine synthetase LanM-like lanthionine synthetase	Thiazolylpeptide Lantibiotic Lantibiotic	24.2 11.0 7.4

A striking feature of secondary metabolism in *H. aurantiacus*, which is also observed in myxobacterial and cyanobacterial genomes, is the preponderance of mixed polyketide synthase (PKS)-non-ribosomal peptide synthetase (NRPS) and NRPS gene clusters [56,57]. On the other hand, only two solely PKS systems were identified, including a type-III PKS of unknown function, as well as an iterative type-I PKS associated with enediyne biosynthesis (Table 6 and Figure 6). Enediyne clusters have as yet only been reported from *Actinobacteria* [58], and the presence of such a locus on the chromosome of *H. aurantiacus* is suggestive of horizontal gene transfer. Evidence for the recent acquisition of biosynthetic genes was obtained in case of the cluster designated *pks-nrps5*. This megacluster, which spans 112.6 kb of contiguous DNA on the chromosome (Table 6), is flanked both upstream and downstream by a number of transposon fragments. An above-average GC content of ~66% as well as significant shifts of the latter in both border regions of the cluster suggest a foreign origin. The observation that horizontal gene transfer may account for the accumulation of biosynthetic genes is, to a degree, reminiscent of the evolution of the *M. xanthus* DK1622 genome [59].

Even though thiotemplate-based chemistry involving PKSs and NRPSs appears to be the predominant theme in natural product biosynthesis by *H. aurantiacus*, genomic analyses also revealed the molecular basis for the assembly and posttranslational modification of ribosomally encoded peptides [60,61]. Furthermore, various pathways to specific biosynthetic building blocks, such as the rare amino acid L-*p*-hydroxyphenylglycine [62] and the polyketide extender unit hydroxymalonyl-acyl carrier protein, were identified. The pathways are located adjacent to PKS and NRPS genes, which suggest that functional crosstalk is very likely to occur.

To date, the secondary metabolome of *Herpetosiphon* spp. has not been explored beyond a single report on a structurally complex, natural product, siphonazole, which likely derives from a mixed PKS-NRPS assembly line [63]. The genome sequence of *H. aurantiacus* strain 114-95^T^ now certainly provides a rationale for further studies of the chemistry and biology of this versatile microorganism.

## References

[1] Holt JG Lewin RA *Herpetosiphon aurantiacus* gen. et sp. n., a new filamentous gliding organism. J Bacteriol 1968; 95:2407-2408 5669912 10.1128/jb.95.6.2407-2408.1968 PMC315177

[2] Skerman VBD McGowan V Sneath PHA Approved Lists of Bacterial Names. Int J Syst Bacteriol 1980; 30:225-420 10.1099/00207713-30-1-225 20806452

[3] Sly LI Taghavi M Fegan M Phylogenetic heterogeneity within the genus *Herpetosiphon*: transfer of the marine species *Herpetosiphon cohaerens, Herpetosiphon nigricans* and *Herpetosiphon persicus* to the genus *Lewinella* gen. nov. in the *Flexibacter*-*Bacteroides*-*Cytophaga* phylum. Int J Syst Bacteriol 1998; 48:731-737 10.1099/00207713-48-3-731 9734027

[4] Euzéby JP List of bacterial names with standing in nomenclature: A folder available on the Internet. Int J Syst Bacteriol 1997; 47:590-592 10.1099/00207713-47-2-590 9103655

[5] Quinn GR Skerman VBD. *Herpetosiphon*—nature’s scavenger? Curr Microbiol 1980; 4:57-62 10.1007/BF02602893

[6] Jurkevitch E Predatory behaviors in bacteria—diversity and transitions. Microbe 2007; 2:67-73

[7] Altschul SF Gish W Miller W Myers EW Lipman DJ Bascic local alignment search tool. J Mol Biol 1990; 215:403-410 2231712 10.1016/S0022-2836(05)80360-2

[8] Korf I, Yandell M, Bedell J. BLAST, O'Reilly, Sebastopol, 2003.

[9] DeSantis TZ Hugenholtz P Larsen N Rojas M Brodie EL Keller K Huber T Dalevi D Hu P Andersen GL Greengenes, a chimera-checked 16S rRNA gene database and workbench compatible with ARB. Appl Environ Microbiol 2006; 72:5069-5072 10.1128/AEM.03006-05 16820507 PMC1489311

[10] Porter MF. An algorithm for suffix stripping. Program: *electronic library and information systems* 1980; **14**:130-137.

[11] Lee C Grasso C Sharlow MF Multiple sequence alignment using partial order graphs. Bioinformatics 2002; 18:452-464 10.1093/bioinformatics/18.3.452 11934745

[12] Castresana J Selection of conserved blocks from multiple alignments for their use in phylogenetic analysis. Mol Biol Evol 2000; 17:540-552 10742046 10.1093/oxfordjournals.molbev.a026334

[13] Stamatakis A Hoover P Rougemont J A rapid bootstrap algorithm for the RAxML web servers. Syst Biol 2008; 57:758-771 10.1080/10635150802429642 18853362

[14] Hess PN De Moraes Russo CA An empirical test of the midpoint rooting method. Biol J Linn Soc Lond 2007; 92:669-674 10.1111/j.1095-8312.2007.00864.x PMC7110036 32287391

[15] Pattengale ND Alipour M Bininda-Emonds ORP Moret BME Stamatakis A How many bootstrap replicates are necessary? Lect Notes Comput Sci 2009; 5541:184-200 10.1007/978-3-642-02008-7_13 20377449

[16] Swofford DL. PAUP*: Phylogenetic Analysis Using Parsimony (*and Other Methods), Version 4.0 b10. Sinauer Associates, Sunderland, 2002.

[17] Liolios K Chen IM Mavromatis K Tavernarakis N Kyrpides NC The genomes on line database (GOLD) in 2009: Status of genomic and metagenomic projects and their associated metadata. Nucleic Acids Res 2010; 38:D346-D354 10.1093/nar/gkp848 19914934 PMC2808860

[18] Wu D Raymond J Wu M Chatterji S Ren Q Graham JE Bryant DA Robb F Colman A Tallon LJ Complete genome sequence of the aerobic CO-oxidizing thermophile *Thermomicrobium roseum.* PLoS ONE 2009; 4:e4207 10.1371/journal.pone.0004207 19148287 PMC2615216

[19] Pati A LaButti K Pukall R Nolan M Glavina Del Rio T Tice H Cheng JF Lucas S Chen F Copeland A Complete genome sequence of *Sphaerobacter thermophilus* type strain (S 6022^T^). Stand Genomic Sci 2010; 2:49-56 10.4056/sigs.601105 21304677 PMC3035262

[20] Field D Garrity G Gray T Morrison N Selengut J Sterk P Tatusova T Thomson N Allen MJ Angiuoli SV The minimum information about a genome sequence (MIGS) specification. Nat Biotechnol 2008; 26:541-547 10.1038/nbt1360 18464787 PMC2409278

[21] Garrity G NamesforLife. BrowserTool takes expertise out of the database and puts it right in the browser. Microbiol Today 2010; 37:9

[22] Woese CR Kandler O Wheelis ML Towards a natural system of organisms. Proposal for the domains *Archaea* and *Bacteria.* Proc Natl Acad Sci USA 1990; 87:4576-4579 10.1073/pnas.87.12.4576 2112744 PMC54159

[23] Hugenholtz P Stackebrandt E Reclassification of *Sphaerobacter thermophilus* from the subclass *Sphaerobacteridae* in the phylum *Actinobacteria* to the class *Thermomicrobia* (emended description) in the phylum *Chloroflexi* (emended description). Int J Syst Evol Microbiol 2004; 54:2049-2051 10.1099/ijs.0.03028-0 15545432

[24] Garrity GM, Holt JG. Phylum BVI. *Chloroflexi* phy. nov. *In*: Garrity GM, Boone DR, Castenholz RW (eds), *Bergey's Manual of Systematic Bacteriology*, Second Edition, Volume 1, Springer, New York, 2001, p. 427-446.

[25] Castenholz RW. Order II. '*Herpetosiphonales*'. *In:* Garrity GM, Boone DR, Castenholz RW (*eds*), *Bergey's Manual of Systematic Bacteriology*, Second Edition, Volume 1, Springer, New York, 2001, p. 444.

[26] Boone DR, Castenholz RW. Family I. '*Herpetosiphonaceae*'. *In:* Garrity GM, Boone DR, Castenholz RW (*eds*), *Bergey's Manual of Systematic Bacteriology*, Second Edition, Volume 1, Springer, New York, 2001, p. 445.

[27] Lewin RA, Leadbetter ER. Genus *Herpetosiphon* Holt and Lewin 1968, 2408; emend. mut. char. In: Buchanan RE, Gibbons NE (eds), *Bergey's Manual of Determinative Bacteriology*, Eighth Edition, The Williams and Wilkins Co., Baltimore, 1974, p. 107-109.

[28] BAuA Classification of bacteria and archaea in risk groups. TRBA 2005; 466:205

[29] Ashburner M Ball CA Blake JA Botstein D Butler H Cherry JM Davis AP Dolinski K Dwight SS Eppig JT Gene ontology: tool for the unification of biology. The Gene Ontology Consortium. Nat Genet 2000; 25:25-29 10.1038/75556 10802651 PMC3037419

[30] Reichenbach H Golecki JR The fine structure of *Herpetosiphon*, and a note on the taxonomy of the genus. Arch Microbiol 1975; 102:281-291 10.1007/BF00428379 1098600

[31] Kleinig H Reichenbach H Carotenoid glucosides and menaquinones from the gliding bacterium *Herpetosiphon giganteus* Hp a2. Arch Microbiol 1977; 112:307-310 10.1007/BF00413098 871230

[32] Jürgen UJ Meißner JD Reichenbach H Weckesser J L-ornithine containing peptidoglycan-polysaccharide complex from the cell wall of the gliding bacterium *Herpetosiphon aurantiacus.* FEMS Microbiol Lett 1989; 60:247-250 10.1016/0378-1097(89)90404-7

[33] Verslyppe B De Smet W De Baets B De Vos P Dawyndt P Make Histri: reconstructing the exchange history of bacterial and archaeal type strains. Syst Appl Microbiol 2011; 34:328-336 10.1016/j.syapm.2011.01.004 21514082

[34] The Joint Genome Institute http://www.jgi.doe.gov/www.jgi.doe.gov

[35] Phrap and Phred for Windows. MacOS, Linux, and Unix. www.phrap.com

[36] Cliff S. Han, Patrick Chain. 2006. Finishing repeat regions automatically with Dupfinisher. Proceeding of the 2006 international conference on bioinformatics & computational biology. Edited by Hamid R. Arabnia & Homayoun Valafar, CSREA Press. June 26-29, 2006: 141-146.

[37] Hyatt D Chen GL LoCascio PF Land ML Larimer FW Hauser LJ Prodigal: prokaryotic gene recognition and translation initiation site identification. BMC Bioinformatics 2010; 11:119 10.1186/1471-2105-11-119 20211023 PMC2848648

[38] Pati A Ivanova NN Mikhailova N Ovchinnikova G Hooper SD Lykidis A Kyrpides NC GenePRIMP: a gene prediction improvement pipeline for prokaryotic genomes. Nat Methods 2010; 7:455-457 10.1038/nmeth.1457 20436475

[39] Markowitz VM Ivanova NN Chen IMA Chu K Kyrpides NC IMG ER: a system for microbial genome annotation expert review and curation. Bioinformatics 2009; 25:2271-2278 10.1093/bioinformatics/btp393 19561336

[40] Bryant DA, Liu Z, Li T, Zhao F, Garcia Costas AM, Klatt CG, Ward DM, Frigaard NU, Overmann J. Comparative and functional genomics of anoxygenic green bacteria from the taxa *Chlorobi, Chloroflexi*, and *Acidobacteria* *In: Advances in Photosynthesis and Respiration*, Vol. 33, *Functional Genomics and Evolution of Photosynthetic Systems*, (Burnap, R. L. and Vermaas, W., eds.), pp. 47-102, Springer, Dordrecht, The Netherlands.

[41] Klatt CG Wood JM Rusch DB Bateson MM Hamamura N Heidelberg JF Grossman AR Bhaya D Cohan FM Kühl M Community ecology of hot spring cyanobacterial mats: predominant populations and their functional potential. ISME J 2011; 5:1262-1278 10.1038/ismej.2011.73 21697961 PMC3146275

[42] Klatt CG Bryant DA Ward DM Comparative genomics provides evidence for the 3-hydroxypropionate autotrophic pathway in filamentous anoxygenic phototrophic bacteria and in hot spring microbial mats. Environ Microbiol 2007; 9:2067-2078 10.1111/j.1462-2920.2007.01323.x 17635550

[43] Maresca JA Bryant DA Identification of two genes encoding new carotenoid-modifying enzymes in the green sulfur bacterium *Chlorobium tepidum.* J Bacteriol 2006; 188:6217-6223 10.1128/JB.00766-06 16923888 PMC1595356

[44] Maresca JA Graham JE Bryant DA Carotenoid biosynthesis in chlorophototrophs: the biochemical and genetic basis for structural diversity. Photosynth Res 2008; 97:121-140 10.1007/s11120-008-9312-3 18535920

[45] Graham JE Bryant DA The biosynthetic pathway for the synthesis of the myxol-2′-fucoside in the cyanobacterium *Synechococcus* sp. strain PCC 7002. J Bacteriol 2009; 191:3292-3300 10.1128/JB.00050-09 19304845 PMC2687168

[46] Takaichi S Maoka T Yamada M Matsuura K Hikawa Y Hanada S Absence of carotenes and presence of a tertiary methoxy group in a carotenoid from a thermophilic filamentous photosynthetic bacterium *Roseiflexus castenholzii.* Plant Cell Physiol 2001; 42:1355-1362 10.1093/pcp/pce172 11773528

[47] Gaitatzis N Kunze B Müller R Novel insights into siderophore formation in myxobacteria. ChemBioChem 2005; 6:365-374 10.1002/cbic.200400206 15678426

[48] Nan B Zusman DR Uncovering the mystery of gliding motility in Myxobacteria. Annu Rev Genet 2011; 45:21-39 10.1146/annurev-genet-110410-132547 21910630 PMC3397683

[49] Luciano J Agrebi R Le Gall AV Wartel M Fiegna F Ducret A Brochier-Armanet C Mignot T Emergence and modular evolution of a novel motility machinery in bacteria. PLoS Genet 2011; 7:e1002268 10.1371/journal.pgen.1002268 21931562 PMC3169522

[50] Rhodes RG Samarasam MN Shrivastava A van Baaren JM Pochiraju S Bollampalli S McBride MJ *Flavobacterium johnsoniae gldN* and *gldO* are partially redundant genes required for gliding motility and surface localization of SprB. [P] J Bacteriol 2010; 192:1201-1211 10.1128/JB.01495-09 20038590 PMC2820869

[51] Rhodes RG Nelson SS Pochiraju S McBride MJ Flavobacterium johnsoniae sprB is part of an operon spanning the additional gliding motility genes *sprC, sprD* and *sprF.* J Bacteriol 2011; 193:599-610 10.1128/JB.01203-10 21131497 PMC3021240

[52] Rhodes RG Pucker HG McBride MJ Development and use of a gene deletion strategy for *Flavobacterium johnsoniae* to identify the redundant gliding motility genes *remF, remG, remH*, and *remI.* J Bacteriol 2011; 193:2418-2428 10.1128/JB.00117-11 21421754 PMC3133171

[53] Rhodes RG Samarasam MN Van Groll EJ McBride MJ Mutations in *Flavobacterium johnsoniae sprE* result in defects in gliding motility and protein secretion. J Bacteriol 2011; 193:5322-5327 10.1128/JB.05480-11 21784937 PMC3187464

[54] Udwary DW Zeigler L Asolkar RN Singan V Lapidus A Fenical W Jensen PR Moore BS Genome sequencing reveals complex secondary metabolome in the marine actinomycete *Salinispora tropica.* Proc Natl Acad Sci USA 2007; 104:10376-10381 10.1073/pnas.0700962104 17563368 PMC1965521

[55] Nett M Ikeda H Moore BS Genomic basis for natural product biosynthetic diversity in the actinomycetes. Nat Prod Rep 2009; 26:1362-1384 10.1039/b817069j 19844637 PMC3063060

[56] Wenzel SC Müller R The impact of genomics on the exploitation of the myxobacterial secondary metabolome. Nat Prod Rep 2009; 26:1385-1407 10.1039/b817073h 19844638

[57] Nett M König GM The chemistry of gliding bacteria. Nat Prod Rep 2007; 24:1245-1261 10.1039/b612668p 18033578

[58] Van Lanen SG Shen B Biosynthesis of enediyne antitumor antibiotics. Curr Top Med Chem 2008; 8:448-459 10.2174/156802608783955656 18397168 PMC3108100

[59] Goldman BS Nierman WC Kaiser D Slater SC Durkin AS Eisen JA Ronning CM Barbazuk WB Blanchard M Field C Evolution of sensory complexity recorded in a myxobacterial genome (2006). Proc Natl Acad Sci USA 2006; 103:15200-15205 10.1073/pnas.0607335103 17015832 PMC1622800

[60] Begley M Cotter PD Hill C Ross RP Identification of a novel two-peptide lantibiotic, lichenicidin, following rational genome mining for LanM proteins. Appl Environ Microbiol 2009; 75:5451-5460 10.1128/AEM.00730-09 19561184 PMC2737927

[61] Brown LCW Acker MG Clardy J Walsh CT Fischbach MA Thirteen posttranslational modifications convert a 14-residue peptide into the antibiotic thiocillin. Proc Natl Acad Sci USA 2009; 106:2549-2553 10.1073/pnas.0900008106 19196969 PMC2650375

[62] Kastner S, Müller S, Natesan L, König GM, Guthke R, Nett M. 4-Hydroxyphenylglycine biosynthesis in *Herpetosiphon aurantiacus* – a case of gene duplication and catalytic divergence. submitted 10.1007/s00203-012-0789-y 22307823

[63] Nett M Erol Ö Kehraus S Köck M Krick A Eguereva E Neu E König GM Siphonazole, an unusual metabolite from *Herpetosiphon* sp. Angew Chem Int Ed Engl 2006; 45:3863-3867 10.1002/anie.200504525 16671154

